# Highlight report: Launch of a large integrated European in vitro toxicology project: EU-ToxRisk

**DOI:** 10.1007/s00204-016-1698-7

**Published:** 2016-03-26

**Authors:** Mardas Daneshian, Hennicke Kamp, Jan Hengstler, Marcel Leist, Bob van de Water

**Affiliations:** Center for Alternatives to Animal Testing - Europe, University of Konstanz, Konstanz, Germany; BASF SE, Ludwigshafen am Rhein, Germany; Leibniz Research Centre for Working Environment and Human Factors, TU Dortmund, Dortmund, Germany; Department of In Vitro Toxicology and Biomedicine, University of Konstanz, Konstanz, Germany; Division of Toxicology, Leiden Academic Center for Drug Research, Leiden University, Leiden, The Netherlands

## Abstract

The integrated European project, EU-ToxRisk, proudly sees itself as “flagship” exploring new alternative-to-animal approaches to chemical safety evaluation. It promotes mechanism-based toxicity testing and risk assessment according to the principles laid down for toxicology for the twenty-first century. The project was officially launched in January 2016 with a kickoff meeting in Egmond aan Zee, the Netherlands. Over 100 scientists representing academia and industry as well as regulatory authorities attended the inaugural meeting. The project will integrate advances in in vitro and in silico toxicology, read-across methods, and adverse outcome pathways. EU-ToxRisk will continue to make use of the case study strategy deployed in SEURAT-1, a FP7 initiative ended in December 2015. Even though the development of new non-animal methods is one target of EU-ToxRisk, the project puts special emphasis on their acceptance and implementation in regulatory contexts. This €30 million Horizon 2020 project involves 38 European partners and one from the USA. EU-ToxRisk aims at the “development of a new way of risk assessment.”

The program intends to drive the required paradigm shift in toxicology toward animal-free, mechanism-based integrated approaches for chemical safety assessment (Leist et al. 2008; NRC 2007; Rovida et al. 2015a, b; Sauer et al. 2015; Scholz et al. 2013). Bennard van Ravenzwaay coordinating the contribution of BASF as a consortium partner pointed out during his inaugural lecture that “this project is rather about regulatory risk assessment than about methods.”

The focus of this 6-year project lies on repeated dose systemic toxicity involving liver, kidney, lung, and the nervous system, as well as on developmental/reproductive toxicity (Hengstler et al. 2012). Particular attention will be paid to the establishment of pragmatic read-across procedures incorporating mechanistic and toxicokinetic knowledge as well as hazard and risk assessment strategies for chemicals with minimal background information (Basketter et al. 2012; Carrio et al. 2015; Patlewicz et al. 2014; van der Burg et al. 2015). EU-ToxRisk will use its resources in order to establish in 3 years’ time a novel read-across approach in Europe, especially fit for evaluating REACH compounds. A quantitatively structured read-across system will use existing data as well as providing new information, including data from high-throughput transcriptomics (Pallocca et al. 2016; Rempel et al. 2015; Grinberg et al. 2014; Jennings et al. 2013; Schaap et al. 2015), high-content imaging of cell stress pathways (Wink et al. 2014; van Vliet et al. 2014; Jennings 2013) in vitro systems (Godoy et al. 2013; Krug et al. 2013), and mathematical modeling to extrapolate to the in vivo situation (Ghallab et al. 2016; Schliess et al. 2014). Moreover, EU-ToxRisk intends to establish a biological read-across approach, adding biological descriptors to toxicological and chemical descriptors (EU-ToxRisk 2015; Zhu et al. 2016). Due to the potential of chemical and biological read-across approaches and the importance of good practice guidelines to this field, EU-ToxRisk’s first workshop on February 26 in Brussels presented the new “Good Read-Across Practice guidance” (Ball et al. 2016) and other relevant initiatives among stakeholders.

EU-ToxRisk coordinator Bob van de Water from the University of Leiden introduced the mission. The overall EU-ToxRisk mission is to develop a quantitative adverse outcome pathway (qAOP) concept for regulatory purposes integrating relevant in vitro and in silico technologies required for the assessment of chemical safety in humans (Leist et al. 2014; Luechtefeld et al. 2015; Muller et al. 2015; Jennings 2013). The concept of EU-ToxRisk was commented by Magdalini Sachana (OECD), an external advisor of the project with the words: “I was delighted to see that adverse outcome pathways (AOPs) and integrated approaches to testing and assessment (IATA) play a central role in almost all the work packages of the project (Tollefsen et al. 2014; Bal-Price et al. 2015; Gocht et al. 2015). I look forward receiving more information and outputs from the project.”

EU-ToxRisk already works on more than ten case studies, and the consortium partners will begin evaluating the predictivity of a battery of assays soon, using data-rich chemicals. Later, case studies will be established for less characterized new classes of compounds. The consortium has already established a task force, which will critically assess the robustness and applicability of the proposed human cell model systems for these case studies. “The case studies with selected compounds will remain focused on adverse human consequences, i.e., provide relevant concentration–response models, and tipping points of homeostasis in order to predict safe exposure levels” as to Carl Westmoreland (Unilever—EU-ToxRisk partner) (Shah et al. 2015). “The importance of building toxicokinetics and toxicodynamics within EU-ToxRisk for weight of evidence approaches in risk assessment” was emphasized by Derek Knight representing ECHA (Berggren et al. 2015; Daston et al. 2015; Gocht et al. 2015). Regarding the case studies, Thomas Steger-Hartmann from Bayer HealthCare commented as an external advisor of the project: “The well-organized and conducted kickoff meeting prepared the stage for an ambitious project that has the potential to change existing safety assessment paradigms. The backbone of the project plan are the case studies. A big part of the success of the project will depend on the thoughtful selection of test compounds, assay systems, and benchmark data for evaluation” (Jennings et al. 2014).

Russell Thomas from the US EPA stated that “I was impressed with the enthusiasm of the scientists involved in the project and the willingness to move beyond basic research and to apply their science to practical, but important questions facing society in testing chemicals for human safety. There appears to be multiple points of intersection between the EU-ToxRisk project and the research being undertaken by the U.S. EPA (Shah et al. 2015; Bouhifd et al. 2015; Kleinstreuer et al. 2014) in the National Center for Computational Toxicology (Liu et al. 2015; Huang et al. 2014). Collaboration at these points of intersection would benefit both organizations and allow us to achieve much more together than in isolation. I look forward to working with and advising the EU-ToxRisk project. It is poised to have a significant impact on the way we evaluate chemicals for human safety.”

The excellent start of the project was helped by extensive preparations by the scientific steering team and the coordinator of the project already in 2015, in order to optimize and refine information flow and work flow of the project. Due to the latter, a remarkable positive and enthusiastic atmosphere during the meeting was notable which is rather exceptional for a kickoff meeting of such a large-scale project. The spirit of all participants of all fourteen work packages was remarkably optimistic and stimulating. If the project keeps up this level of dynamics and thrives, a lot is to be expected for the near future and the coming 6 years.

## References

[CR1] Ball N, Cronin MT, Shen J (2016). Toward good read-across practice (GRAP) guidance. ALTEX.

[CR2] Bal-Price A, Crofton KM, Leist M, Allen S, Arand M, Buetler T, Delrue N, FitzGerald RE, Hartung T, Heinonen T, Hogberg H, Bennekou SH, Lichtensteiger W, Oggier D, Paparella M, Axelstad M, Piersma A, Rached E, Schilter B, Schmuck G, Stoppini L, Tongiorgi E, Tiramani M, Monnet-Tschudi F, Wilks MF, Ylikomi T, Fritsche E (2015). International STakeholder NETwork (ISTNET): creating a developmental neurotoxicity (DNT) testing road map for regulatory purposes. Arch Toxicol.

[CR3] Basketter DA, Clewell H, Kimber I (2012). A roadmap for the development of alternative (non-animal) methods for systemic toxicity testing—t4 report*. ALTEX.

[CR4] Berggren E, Amcoff P, Benigni R (2015). Chemical safety assessment using read-across: assessing the use of novel testing methods to strengthen the evidence base for decision making. Environ Health Perspect.

[CR5] Bouhifd M, Andersen ME, Baghdikian C, Boekelheide K, Crofton KM, Fornace AJ, Kleensang A, Li H, Livi C, Maertens A, McMullen PD, Rosenberg M, Thomas R, Vantangoli M, Yager JD, Zhao L, Hartung T (2015). The human toxome project. ALTEX.

[CR6] Carrio P, Sanz F, Pastor M (2015). Toward a unifying strategy for the structure-based prediction of toxicological endpoints. Arch Toxicol.

[CR7] Daston G, Knight DJ, Schwarz M (2015). SEURAT: Safety evaluation ultimately replacing animal testing—recommendations for future research in the field of predictive toxicology. Arch Toxicol.

[CR8] EU-ToxRisk (2015). EU: EU-ToxRisk to start in 2016. ALTEX.

[CR9] Ghallab A, Cellière G, Henkel SG, Driesch D, Hoehme S, Hofmann U, Zellmer S, Godoy P, Sachinidis A, Blaszkewicz M, Reif R, Marchan R, Kuepfer L, Häussinger D, Drasdo D, Gebhardt R, Hengstler JG (2016). Model-guided identification of a therapeutic strategy to reduce hyperammonemia in liver diseases. J Hepatol.

[CR10] Gocht T, Berggren E, Ahr HJ (2015). The SEURAT-1 approach towards animal free human safety assessment. ALTEX.

[CR11] Godoy P, Hewitt NJ, Albrecht U (2013). Recent advances in 2D and 3D in vitro systems using primary hepatocytes, alternative hepatocyte sources and non-parenchymal liver cells and their use in investigating mechanisms of hepatotoxicity, cell signaling and ADME. Arch Toxicol.

[CR12] Grinberg M, Stöber RM, Edlund K, Rempel E, Godoy P, Reif R, Widera A, Madjar K, Schmidt-Heck W, Marchan R, Sachinidis A, Spitkovsky D, Hescheler J, Carmo H, Arbo MD, van de Water B, Wink S, Vinken M, Rogiers V, Escher S, Hardy B, Mitic D, Myatt G, Waldmann T, Mardinoglu A, Damm G, Seehofer D, Nüssler A, Weiss TS, Oberemm A, Lampen A, Schaap MM, Luijten M, van Steeg H, Thasler WE, Kleinjans JC, Stierum RH, Leist M, Rahnenführer J, Hengstler JG (2014). Toxicogenomics directory of chemically exposed human hepatocytes. Arch Toxicol.

[CR13] Hengstler JG, Marchan R, Leist M (2012). Highlight report: towards the replacement of in vivo repeated dose systemic toxicity testing. Arch Toxicol.

[CR14] Huang R, Sakamuru S, Martin MT, Reif DM, Judson RS, Houck KA, Casey W, Hsieh JH, Shockley KR, Ceger P, Fostel J, Witt KL, Tong W, Rotroff DM, Zhao T, Shinn P, Simeonov A, Dix DJ, Austin CP, Kavlock RJ, Tice RR, Xia M (2014). Profiling of the Tox21 10K compound library for agonists and antagonists of the estrogen receptor alpha signaling pathway. Sci Rep.

[CR15] Jennings P (2013). Stress response pathways, toxicity pathways and adverse outcome pathways. Arch Toxicol.

[CR16] Jennings P, Limonciel A, Felice L, Leonard MO (2013). An overview of transcriptional regulation in response to toxicological insult. Arch Toxicol.

[CR17] Jennings P, Schwarz M, Landesmann B, Maggioni S, Goumenou M, Bower D, Leonard MO, Wiseman JS (2014). SEURAT-1 liver gold reference compounds: a mechanism-based review. Arch Toxicol.

[CR18] Kleinstreuer NC, Yang J, Berg EL, Knudsen TB, Richard AM, Martin MT, Reif DM, Judson RS, Polokoff M, Dix DJ, Kavlock RJ, Houck KA (2014). Phenotypic screening of the ToxCast chemical library to classify toxic and therapeutic mechanisms. Nat Biotechnol.

[CR19] Krug AK, Kolde R, Gaspar JA, Rempel E, Balmer NV, Meganathan K, Vojnits K, Baquié M, Waldmann T, Ensenat-Waser R, Jagtap S, Evans RM, Julien S, Peterson H, Zagoura D, Kadereit S, Gerhard D, Sotiriadou I, Heke M, Natarajan K, Henry M, Winkler J, Marchan R, Stoppini L, Bosgra S, Westerhout J, Verwei M, Vilo J, Kortenkamp A, Hescheler J, Hothorn L, Bremer S, van Thriel C, Krause KH, Hengstler JG, Rahnenführer J, Leist M, Sachinidis A (2013). Human embryonic stem cell-derived test systems for developmental neurotoxicity: a transcriptomics approach. Arch Toxicol.

[CR20] Leist M, Hartung T, Nicotera P (2008). The dawning of a new age of toxicology. ALTEX.

[CR21] Leist M, Hasiwa N, Rovida C (2014). Consensus report on the future of animal-free systemic toxicity testing. ALTEX.

[CR22] Liu J, Mansouri K, Judson RS (2015). Predicting hepatotoxicity using ToxCast in vitro bioactivity and chemical structure. Chem Res Toxicol.

[CR23] Luechtefeld T, Maertens A, McKim JM, Hartung T, Kleensang A, Sa-Rocha V (2015). Probabilistic hazard assessment for skin sensitization potency by dose-response modeling using feature elimination instead of quantitative structure-activity relationships. J Appl Toxicol.

[CR24] Muller EB, Lin S, Nisbet RM (2015). Quantitative adverse outcome pathway analysis of hatching in zebrafish with CuO nanoparticles. Environ Sci Technol.

[CR25] NRC (2007) Toxicity testing in the 21st century: a vision and a strategy. Washington, DC

[CR26] Pallocca G, Grinberg M, Henry M (2016). Identification of transcriptome signatures and biomarkers specific for potential developmental toxicants inhibiting human neural crest cell migration. Arch Toxicol.

[CR27] Patlewicz G, Ball N, Becker RA (2014). Read-across approaches—misconceptions, promises and challenges ahead. ALTEX.

[CR28] Rempel E, Hoelting L, Waldmann T (2015). A transcriptome-based classifier to identify developmental toxicants by stem cell testing: design, validation and optimization for histone deacetylase inhibitors. Arch Toxicol.

[CR29] Rovida C, Alepee N, Api AM (2015). Integrated testing strategies (ITS) for safety assessment. ALTEX.

[CR30] Rovida C, Asakura S, Daneshian M (2015). Toxicity testing in the 21st century beyond environmental chemicals. ALTEX.

[CR31] Sauer JM, Hartung T, Leist M, Knudsen TB, Hoeng J, Hayes AW (2015). Systems toxicology: the future of risk assessment. Int J Toxicol.

[CR32] Schaap MM, Wackers PF, Zwart EP, Huijskens I, Jonker MJ, Hendriks G, Breit TM, van Steeg H, van de Water B, Luijten M (2015). A novel toxicogenomics-based approach to categorize (non-)genotoxic carcinogens. Arch Toxicol.

[CR33] Schliess F, Hoehme S, Henkel SG, Ghallab A, Driesch D, Böttger J, Guthke R, Pfaff M, Hengstler JG, Gebhardt R, Häussinger D, Drasdo D, Zellmer S (2014). Integrated metabolic spatial-temporal model for the prediction of ammonia detoxification during liver damage and regeneration. Hepatology.

[CR34] Scholz S, Sela E, Blaha L (2013). A European perspective on alternatives to animal testing for environmental hazard identification and risk assessment. Regul Toxicol Pharmacol.

[CR35] Shah I, Setzer RW, Jack J, Houck KA, Judson RS, Knudsen TB, Liu J, Martin MT, Reif DM, Richard AM, Thomas RS, Crofton KM, Dix DJ, Kavlock RJ (2015) Using ToxCast™ data to reconstruct dynamic cell state trajectories and estimate toxicological points of departure. Environ Health Perspect. doi:10.1289/ehp.1409029 10.1289/ehp.1409029 PMC4937847 26473631

[CR36] Tollefsen KE, Scholz S, Cronin MT (2014). Applying adverse outcome pathways (AOPs) to support integrated approaches to testing and assessment (IATA). Regul Toxicol Pharmacol.

[CR37] van der Burg B, Wedebye EB, Dietrich DR (2015). The ChemScreen project to design a pragmatic alternative approach to predict reproductive toxicity of chemicals. Reprod Toxicol.

[CR38] van Vliet E, Daneshian M, Beilmann M, Davies A, Fava E, Fleck R, Julé Y, Kansy M, Kustermann S, Macko P, Mundy WR, Roth A, Shah I, Uteng M, van de Water B, Hartung T, Leist M (2014). Current approaches and future role of high content imaging in safety sciences and drug discovery. ALTEX.

[CR39] Wink S, Hiemstra S, Huppelschoten S, Danen E, Niemeijer M, Hendriks G, Vrieling H, Herpers B, van de Water B (2014). Quantitative high content imaging of cellular adaptive stress response pathways in toxicity for chemical safety assessment. Chem Res Toxicol.

[CR40] Zhu H, Bouhifd M, Kleinstreuer N (2016). Supporting read-across using biological data. ALTEX.

